# Association of Patient and Neighborhood Factors With Lung Cancer Screening Rates in Minnesota

**DOI:** 10.1016/j.chpulm.2025.100228

**Published:** 2025-11-17

**Authors:** Milind Bhagat, Rebecca Freese, David Haynes, Abbie Begnaud

**Affiliations:** a Division of Pulmonary, Allergy, Critical Care and Sleep, Department of Medicine, University of Minnesota, Minneapolis, MN; b Clinical and Translational Science Institute, Biostatistical Design and Analysis Center, University of Minnesota, Minneapolis, MN; c Institutes for Health Informatics, Department of Medicine, University of Minnesota, Minneapolis, MN

**Keywords:** low-dose CT scan, lung cancer screening, screening disparities

## Abstract

**Background:**

Lung cancer is the second most common cancer and the leading cause of cancer deaths. Despite the known benefits of lung cancer screening, it is estimated to have low population uptake due to a variety of factors. We sought to estimate lung cancer screening rates in a large health system in Minnesota and study the factors affecting completion.

**Research Question:**

What are the rates of lung cancer screening chest CT scan ordered and completed in eligible populations in Minnesota’s large integrated health system and its association with various socioeconomic factors?

**Study Design and Methods:**

We extracted records of patients likely eligible for lung cancer screening based on age and smoking history and determined their screening completion rates. Logistic regression models were used to evaluate associations. ORs and 95% CIs were calculated and evaluated at *P* = .05 for statistical significance.

**Results:**

Approximately 37% of patients with a smoking history were eligible for lung cancer screening. Approximately 12% of these patients had any CT chest scan completed, with only 7.2% being a screening-intent low-dose CT (LDCT) scan. The odds of screening were more strongly associated with age, smoking status, and gender than state Area Deprivation Index decile. Patients who previously smoked had twice the odds of having an LDCT scan order and greater odds of completion. People ≥ 65 years of age were more likely to have an order and completion than younger patients. Men had slightly lower odds of having an LDCT scan order than women, but they had greater odds of completion.

**Interpretation:**

Our results suggest that the uptake of lung cancer screening continues to be low. Older patients, male individuals, and those who quit smoking were shown to be more likely to have a screening LDCT scan completed with no significant effect of state Area Deprivation Index decile.

**Take-Home Points**

**Study Question:** What are the rates of lung cancer screening in a large academic health care system of Minnesota and what are the different factors affecting its completion?

**Results:** Approximately 7% of eligible patients had a screening-intent low-dose CT scan completed. The odds of screening were more strongly associated with age, smoking status, and gender than state Area Deprivation Index decile.

**Interpretation:** This study showed that the real-world rates of lung cancer screening completion among the eligible patient population continue to be low with various patient-level factors affecting it.

Although lung cancer is the second most common cancer (behind prostate cancer in male patients and breast cancer in female patients), it claims more lives than breast, prostate, and colon cancer combined (> 20%).^1^ Current (2021) US Preventive Services Task Force (USPSTF)^2^ guidelines recommend lung cancer screening (LCS) with low-dose CT (LDCT) in adults 50 to 80 years of age who have a 20-pack-year smoking history, and currently smoke or have quit within the past 15 years. Long-term follow-up of early screening trials^3^ shows 81% lung cancer-specific survival at 10 and 20 years. However, uptake has been slow. Estimating LCS uptake is challenging because the number of eligible people is unknown, with 1 population-based study estimating the number to be around 13 million.^4^ Patient-level data from health systems is inaccurate because quantitative smoking history is often incomplete.^5^ Most estimates are based on national health surveys but are not linked to health records to confirm LDCT scan completion. The American Lung Association’s State of Lung Cancer 2022 report^6^ estimated that only 5.8% of those at high risk of lung cancer were screened nationally.

Various reasons responsible for low uptake of the screening guidelines have been described in the literature, which can be broadly divided into the following 3 categories: (1) individual patient-level factors, which can include cognitive, psychological, or access-related issues^7^; (2) health care system factors, which include lack of provider knowledge, comorbid medical conditions impacting risk-benefit balance, or disparate distribution of screening services; and (3) social determinants of health (SDoH).^8^ The World Health Organization defines SDoH as the nonmedical factors influencing health outcomes: “conditions in which people are born, grow, work, live, and age, and the wider set of forces and systems shaping the conditions of daily life.”^9^ The role of SDoH in screening uptake has been studied in many cancers, including lung.^10^, ^11^, ^12^ Korn et al^10^ divided these factors into 4 broad categories: health care access and quality, education access and quality, social and community context, and neighborhood and built environment. The domain with the most potential for impactful interventions was proposed to be the neighborhood and built environment (including transportation). Understanding these factors’ role could help develop more interventions to mitigate them, leading to more equitable health outcomes.

Multiple studies have shown that socioeconomic deprivation is associated with higher lung cancer prevalence, higher mortality, and poor survival,^13^, ^14^, ^15^, ^16^ which is primarily due to diagnosis at a later stage^17^^,^^18^; thus, improving the uptake of LCS in individuals experiencing socioeconomic deprivation may be particularly impactful. An effective index to measure SDoH is the Area Deprivation Index (ADI), first constructed by Singh^19^ in 2003. It has since been refined, adapted, and validated to the Census Block Group neighborhood level by Kind et al^20^ at the University of Wisconsin-Madison. It allows for rankings of neighborhoods by socioeconomic disadvantage in a region of interest (eg, at the state or national level). It incorporates factors for the theoretical domains of income, education, employment, and housing quality.

In this study, we set out to estimate rates of LCS by USPSTF 2021 recommendations using patient-level data of an integrated health system in Minnesota and to examine factors associated with LCS completion.

## Study Design and Methods

We collaborated with the University of Minnesota Best Practices Integrated Informatics Core to extract records of patients who might be eligible for LCS and completion rates for chest imaging for those patients from the Clarity database of our health system electronic heath record (EHR), EPIC. The cohort was identified in October 2019 to 2022 and included potentially LCS-eligible patients: those 50 to 80 years of age with any recorded history of cigarette smoking and who had been seen in a primary care clinic (internal medicine or family medicine) in the last 3 years. Patients who had opted out of research consideration were excluded as were any patients who were reported to have never smoked cigarettes. Each participant’s home address was geocoded and spatially joined to the 2020 census block, and the 2020 Minnesota ADI decile for each census block was assigned (2020 ADI values are derived from the 2016 to 2020 American Community Survey). For all included participants, we extracted chest CT scan completion as of October 19, 2022, including screening LDCT scan. All elements of smoking history recorded in the substance and social history were also included in the extracted report. Smoking history was considered sufficient to determine eligibility if a patient was reported as currently smoking or if individuals who quit smoking had quit year, number of years smoked, and packs per day smoked entered to enable calculation of pack-years. We classified patients as likely eligible if they were reported to be currently smoking or if they had quit smoking and had sufficient complete smoking history satisfying USPSTF LCS eligibility criteria (quit time < 15 years and at least 20 pack-years reported). Patients with indeterminate eligibility (former smoking status but without a quit date, pack-years, or both) were excluded. LDCT scan orders were characterized as screening-intended if the ordering clinician used a unique imaging order for LCS. Screening-intent LDCT scan was designated by the Current Procedural Terminology codes G0297 or 71270 or 71271 (after January 31, 2021). Participant completion of other chest CT procedures was also recorded, including diagnostic chest CT scan with or without contrast (Current Procedural Terminology codes 71250, 71260, or 71270).

Participant characteristics were summarized with mean ± SD for continuous variables and count (%) for categorical variables. Logistic regression models were used to evaluate associations between having an LDCT screening ordered, completed, or any other LDCT scan completed and state ADI and sex. A sensitivity analysis was performed by repeating the analyses including the patients whose smoking history documentation was insufficient to determine LCS eligibility. This sensitivity analysis was performed because a major limitation to EHR research on LCS is the frequency of incomplete smoking history documentation. The main outcomes were assessed on patients deemed likely to be eligible by EHR smoking history, but for the sensitivity analysis, we analyzed the outcomes in patients whose smoking history was such that LCS eligibility was indeterminate but possible, such as those with a former smoking status and no quit date. Models were adjusted for age category and smoking status. ORs and 95% CIs were calculated and evaluated at .05 for statistical significance. *P* values are 2-sided, and all analyses were completed in R (R Core Team, 2023), version 4.3.2. The study was determined by the local institutional review board to be exempt from review.

## Results

A total of 248,952 patients were in the cohort of adults 50 to 80 years of age seen in primary care clinics with any smoking history, and 92,446 (37%) were likely eligible for LCS (Table 1). Of the patients, 49.2% were female with only 4 missing sex data. Most of the patients (85.2%) were White; 6.7% were Black. There were 1,194 (1.2%) and 881 patients (< 1%) with missing data for race and preferred language, respectively. More than 98% of the patients had sufficient information to ascertain their eligibility.

Approximately 12% of likely eligible people had any chest CT scan completed in our health care system (ie, CT scan intended for screening or another chest CT scan), but only 7.2% had a screening-intent LDCT scan completed (Table 2). We analyzed association of screening orders and completion rates with different variables including age, sex, smoking status, and ADI.

The odds of screening were more strongly associated with age, smoking status, and gender than state ADI decile (Tables 3, 4). People ≥ 65 years of age had > 2 times the odds of having an order for LDCT scan than younger participants and 40% higher odds of completing LDCT scan when an order was in place. People who quit smoking had 2 times the odds of having an LDCT scan order and greater odds of completing the LDCT scan with an order in place compared with participants with current smoking status. Male individuals had slightly lower odds of having an LDCT scan order than female individuals, but they had greater odds of completing it when ordered. All relationships were consistent but weaker when looking at the completion of any LDCT scan, including those not intended for LCS. Sensitivity analysis conducted by including these indeterminate eligibility participants did not show a difference in any odds from Tables 3 and 4.

**Table 1:** Participant Characteristics, Likely Eligible for Lung Cancer Screening and Seen in Primary Care Clinics in the Last 3 Years (N = 92,446)

Characteristic	Value
Sex	
Female	45,494 (49.2)
Male	46,948 (50.8)
Missing, No.	4
Race	
American Indian or Alaska Native	1,197 (1.3)
Asian	1,609 (1.8)
Black	6,131 (6.7)
Choose not to answer	3,494 (3.8)
Middle Eastern	40 (0.0)
Native Hawaiian or other Pacific Islander	107 (0.1)
White	77,741 (85.2)
> 1 race	933 (1.0)
Missing	1,194 (1.2)
Preferred language	
English	89,645 (97.9)
Non-English	1,920 (2.1)
Missing, No.	881
Classification of recorded smoking history	
Sufficient: current smoking status	74,883 (81.0)
Sufficient: former smoking status + quit year + pack-years	16,121 (17.4)
Partial: former smoking status + quit date; no pack-years	1,442 (1.6)
ADI state	
Mean [SD] (missing)	5.4 [2.7] (5,417)

Data are presented as No. (%) or as otherwise indicated. All percentages are calculated out of nonmissing values. ADI = Area Deprivation Index.

**Table 2:** Lung Cancer Screening Rates Among Likely Eligible Participants (N = 92,446)

Variable Value	
Screening-intent LDCT scan ordered	
No	83,435 (90.3)
Yes	9,011 (9.7)
Screening-intent LDCT scan completed	
No	85,816 (92.8)
Yes	6,630 (7.2)
Non-screening-intent chest CT scan completed	
No	87,611 (94.8)
Yes	4,835 (5.2)

LDCT = low-dose CT.

**Table 3:** Odds of Screening: Logistic Regression ADI and Lung Cancer Screening Outcomes

Term	OR	95% CI	*P* Value
Screening-intent LDCT scan ordered, likely eligible			
State ADI	1.02	1.01-1.03	< .001
Age: ≥ 65+ vs < 65 y	2.53	2.41-2.64	< .001
Smoking status: former vs current	1.93	1.83-2.03	< .001
Screening-intent LDCT scan completed, likely eligible (with LDCT scan order)			
State ADI	1.00	0.98-1.02	.875
Age: ≥ 65 vs < 65 y	1.39	1.27-1.54	< .001
Smoking status: former vs current	1.44	1.30-1.60	< .001
Any chest CT scan completed, likely eligible (with LDCT scan order)			
State ADI	0.95	0.93-0.98	< .001
Age: ≥ 65 vs < 65 y	1.56	1.37-1.78	< .001
Smoking status: former vs current	1.29	1.13-1.47	< .001

ADI = Area Deprivation Index; LDCT = low-dose CT.

**Table 4:** Odds of Screening: Logistic Regression Sex and Lung Cancer Screening Outcomes

Term	OR	95% CI	*P* Value
Ordered, likely eligible			
Sex: male vs female	0.87	0.83-0.90	< .001
Age: ≥ 65 vs < 65 y	2.51	2.40-2.62	< .001
Smoking status: former vs current	1.91	1.81-2.00	< .001
LDCT scan completed, likely eligible (with LDCT scan order)			
Sex: male vs female	1.18	1.07-1.30	< .001
Age: ≥ 65 vs < 65 y	1.40	1.27-1.54	< .001
Smoking status: former vs current	1.44	1.30-1.60	< .001
Any chest CT scan completed, likely eligible (with LDCT scan order)			
Sex: male vs female	0.91	0.81-1.04	.160
Age: ≥ 65 vs < 65 y	1.60	1.40-1.81	< .001
Smoking status: former vs current	1.31	1.15-1.49	< .001

LDCT = low-dose CT.

## Discussion

Current USPSTF guidelines recommend LCS in eligible populations with annual LDCT chest scan. This has been shown to reduce mortality through stage shift and early detection. Despite the moderate net benefit noted by the USPSTF in their LCS recommendations, significant opportunities remain at the patient, population, and health care system level to improve low uptake of LCS. This study estimated current rates of USPSTF-recommended LCS in a large population of an integrated health system in Minnesota, which were found to be very low (approximately 11%).

The screening rates found in our study are slightly lower than recently published LCS prevalence data nationally and in Minnesota by Henderson et al.^21^ That study used Behavioral Risk Factor Suveillance System data for 2022 and used self-reported smoking history. Henderson et al^21^ estimated LCS uptake closer to 17% in Minnesota, and our system-wide numbers may be diluted by including patients with any primary care visit in the last 3 years, considering some of those may have been seen for acute illness (eg, COVID-19 testing or treatment) while seeking preventive care and wellness visits elsewhere. Another explanation for the discrepancy between our study and Henderson et al^21^ is that Behavioral Risk Factor Suveillance System data rely on participant self-report of LCS completion. Our findings are close to the rates estimated during the COVID-19 pandemic by Fedewa et al.^20^

There was a statistically significant but a negligible relationship between the ADI and odds of LDCT scan order and completion, but factors such as age, smoking status, and gender were associated more strongly. Patients ≥ 65 years of age were more than twice as likely to have LDCT scan ordered and about 1.5 times more likely to have it completed than patients < 65 years of age. Our findings that people who quit smoking are more likely to be screened are also like prior reports.^22^ In the Silvestri et al^23^ study describing the characteristics of > 1 million LDCT scans between 2015 and 2019 from the Lung Cancer Screening Registry (LCSR), these were compared with 1,257 respondents from the 2015 National Health Interview Survey (NHIS). In this study, people screened were more likely to be female, ≥ 65 years of age, and currently smoke than the eligible population among the NHIS respondents. The increased likelihood of male individuals to complete LDCT scan (when an order is placed) may not be inconsistent with Silvestri et al^23^ because neither the NHIS nor the LCSR have data on LDCT orders that were placed but never completed. That is to say that the NHIS and LCSR are unable to determine who had orders for LCS that were never completed. A unique strength of our study is EHR data of unrealized LCS (signed orders never completed). One other possible explanation for the discordant findings between our single-site study using patient-level data and their study is the use of the NHIS, which may have limitations in determining true LCS uptake related to inaccurate self-reporting of LDCT scan completion. Survey respondents may incorrectly recall LDCT scan for LCS completion due to confusion with other types of testing or CT imaging for other indications (nonscreening CT scans). Considering only the population screened under the LCSR in this study, patients screened were more likely to be male and ≥ 65 years of age, which would be concordant to our findings. Similar results were also found in the study by Pinsky and Miller,^24^ with screening rates higher in male individuals than female individuals.

We chose the ADI as an alternative measure of SDoH and social deprivation, but it has previously been correlated with race. A previous study did show a difference in cancer screening completion between the most and least deprived quintiles using the ADI. However, that analysis derived modified ADI values using factor analysis of the individual variables that contribute to the ADI, including weighting and standardization. In our study, the patient population studied did not have a normal distribution of ADI, thus limiting our ability to detect a signal.

These findings mean that there is an outsized missed opportunity to increase LCS rates through easier identification of unscreened eligible people. Patients who currently smoke in the 50- to-80-year age range are quite likely to be eligible.^25^ Interestingly, although male patients had lower statistical odds of having LDCT scan ordered, they were more likely than females to complete the CT scan. Perhaps this represents a known phenomenon of healthy/asymptomatic men being less likely to conduct preventive care visits.^26^ Participants reporting former cigarette smoking were more likely to have the LDCT scan ordered and completed compared with those reporting current smoking. This represents an opportunity for outreach and destigmatization of cigarette smoking, along with empathetic support to quit or cut back. Although the minimal impact of ADI disparity in LDCT scans ordered and completed might be a result of a variety of state-level systems, such as low rates of uninsured people in Minnesota, the results suggest that for patients engaging in primary care visits, neighborhood deprivation is less influential than other factors for completing LCS.

## Interpretation

The uptake of LCS, among likely eligible patients, as measured through individual patient-level data, continues to be low at 7%. Patients ≥ 65 years of age, of male gender and who quit smoking, were more likely to have a screening LDCT scan completed with no significant effect of state ADI decile. These findings can be used to devise future interventions targeted at increasing LCS rates.

### Strengths of the Study

To our knowledge, this is 1 of the few studies to report real-world rates of confirmed LCS in a large population of eligible patients using patient-level EHR data, comparing LDCT scan orders and completion. This study also examines the impact of age, sex, and smoking status on LCS orders and completion rates.

### Limitations of the Study

The cross-sectional study design prevents any causal inference on the associations observed. Because the data are limited to a single large health system, it may not be applicable to the general screening population. In Minnesota, uninsured rates are very low,^27^^,^^28^ like other states with Medicaid expansion. In states with higher rates of uninsurance, the ADI might be a more predictive factor for LCS outcomes.

## Funding/Support

This research was supported by the National Institutes of Health’s National Center for Advancing Translational Sciences [Grant UM1TR004405].

## Financial/Nonfinancial Disclosures

None declared.
